# Investigation of the Profile Control Mechanisms of Dispersed Particle Gel

**DOI:** 10.1371/journal.pone.0100471

**Published:** 2014-06-20

**Authors:** Guang Zhao, Caili Dai, Mingwei Zhao

**Affiliations:** State Key Laboratory of Heavy Oil Processing, China University of Petroleum, Qingdao, Shandong, P. R. China; University of California San Diego, United States of America

## Abstract

Dispersed particle gel (DPG) particles of nano- to micron- to mm-size have been prepared successfully and will be used for profile control treatment in mature oilfields. The profile control and enhanced oil recovery mechanisms of DPG particles have been investigated using core flow tests and visual simulation experiments. Core flow test results show that DPG particles can easily be injected into deep formations and can effectively plug the high permeability zones. The high profile improvement rate improves reservoir heterogeneity and diverts fluid into the low permeability zone. Both water and oil permeability were reduced when DPG particles were injected, but the disproportionate permeability reduction effect was significant. Water permeability decreases more than the oil permeability to ensure that oil flows in its own pathways and can easily be driven out. Visual simulation experiments demonstrate that DPG particles can pass directly or by deformation through porous media and enter deep formations. By retention, adsorption, trapping and bridging, DPG particles can effectively reduce the permeability of porous media in high permeability zones and divert fluid into a low permeability zone, thus improving formation profiles and enhancing oil recovery.

## Introduction

Oil recovery occurs through three main processes: primary, secondary, and tertiary recovery [1]. Primary recovery refers to the volume of oil produced by the natural energy available in the reservoir. However, only approximately 10–20 percent of the original oil is typically produced during primary recovery. Once the natural reservoir energy has been depleted, and the well oil production rates decline during primary recovery, additional energy to maintain the formation pressure is necessary [2]. Generally, water is injected into oil reservoirs to complement or increase the original energy within the reservoir. This process is called secondary recovery or water flooding injection. Secondary oil recovery commonly ranges from 20% to 40% [3]. However, long term water flooding during the development of the oilfield has resulted in the aggravated heterogeneity of reservoirs, thus severely affecting the flow of gas, oil and water in the reservoir. The change in flow results in the breakthrough of the injection water into producing wells along the high permeability channels or fractures, thereby reducing oil production. So improving formation heterogeneity and minimizing water use becomes an important emergency objective for mature oilfields. Many technologies have been applied successfully, including injection polymers, gelling systems, particle systems, and foams, etc. [4]–[11]. However, a large amount of polymers are used in polymer flooding, which greatly increases recovery costs. In addition, polymer flooding is not suitable for serious heterogeneous reservoirs because of its weak profile control and water shut-off capability. Gel systems have inherent drawbacks such as uncontrolled gelation time, uncertainty of gelling due to shear degradation, changes in gel composition, and dilution caused by contact with reservoir minerals and fluids. Moreover, the foam injection technique has a short validity because of unsustainable nitrogen or air sources.

To overcome these problems, particle systems were synthesized in surface facilities [12]–[17]. Among these particle systems, DPG particles for profile control have attracted great interest in recent years. DPG particles have excellent properties, such as adjustable size distribution from nm to mm, thermal stability, and shearing resistance, etc., for profile control treatment. Generally, two methods are available for the preparation of DPG particles: the shearing cross-linking method using a coaxial cylinder viscometer or peristaltic pump or the high speed shearing method using a colloid mill. Both of the two methods involve shearing forces, but there is an important difference in the preparation mechanism. The shearing forces generated from the coaxial cylinder viscometer or peristaltic pump are conducted to the gelling solution, which creates a discontinuous gel system and forms DPG particles. However, the shearing forces generated from the colloid mill are directly conducted to the bulk gel, which changes the bulk gel into small particles and forms DPG particles. Due to the smaller displacement and lower production efficiency of the coaxial cylinder viscometer or peristaltic pump, the viscometer or peristaltic pump cannot meet the requirements for large-scale production, which limits the development of a technology application for the oilfield. For easy preparation, easy injection and industry demands, DPG particles are prepared using cross-linked gel systems through a high speed shearing method at room temperature. Although DPG particles have been prepared successfully, little research has been conducted on the profile control mechanism of the particles. To better apply the DPG particles in oilfields, core flow tests and visual simulation experiments were performed to investigate the profile control mechanisms of the particles. We primarily studied the injection capacity, profile improvement capacity, disproportionate permeability reduction capacity of water and oil, and propagation and plug patterns of the DPG particles in porous media. Additionally, a detailed profile control mechanism was also discussed. The results of this study may serve as a reference for understanding the profile control mechanisms of DPG particles. Through laboratory experiments, we expect that the work can be further promoted and become applicable for profile control in mature oilfields.

## Materials and Methods

### 2.1 Materials

Nonionic polyacrylamide (PAM) with a degree of hydrolysis of 3.31% and average molecular weight of 9,650,000 g/mol was provided by Yuguang Co., Ltd., China. Zirconium acetate as a crosslinker was purchased from Zibo Co., Ltd., China. The brine salinity used in all experiments was 492.08 mg/L.

### 2.2 Preparation of DPG particles

The entire preparation process for DPG particles was divided into two successive steps [17]: the bulk gel crosslinking reaction period and the DPG particle preparation period. In the bulk gel crosslinking reaction period, gel solution was prepared by mixing polymer solution and crosslinker at room temperature [18]. The polymer solution was first diluted to the required concentration using brine. Crosslinker was then dropped slowly into the polymer solution and stirred to produce a uniform gel solution. In this study, a typical gel formulation contains 0.6% polymer and 1.6% crosslinker, and the crosslinking reaction lasts for approximately 43 minutes at 30°C. When the bulk gel was formed, 200 g of brine water and 200 g of bulk gel were added simultaneously to a colloid mill (JM-85 type, Shandong Longxing Instruments, Ltd., China) rotating at 1000–3000 rpm and milled for 3–15 min at room temperature. The solution obtained was the final DPG product.

### 2.3 Determination of injection capacity

**Figure 1:**
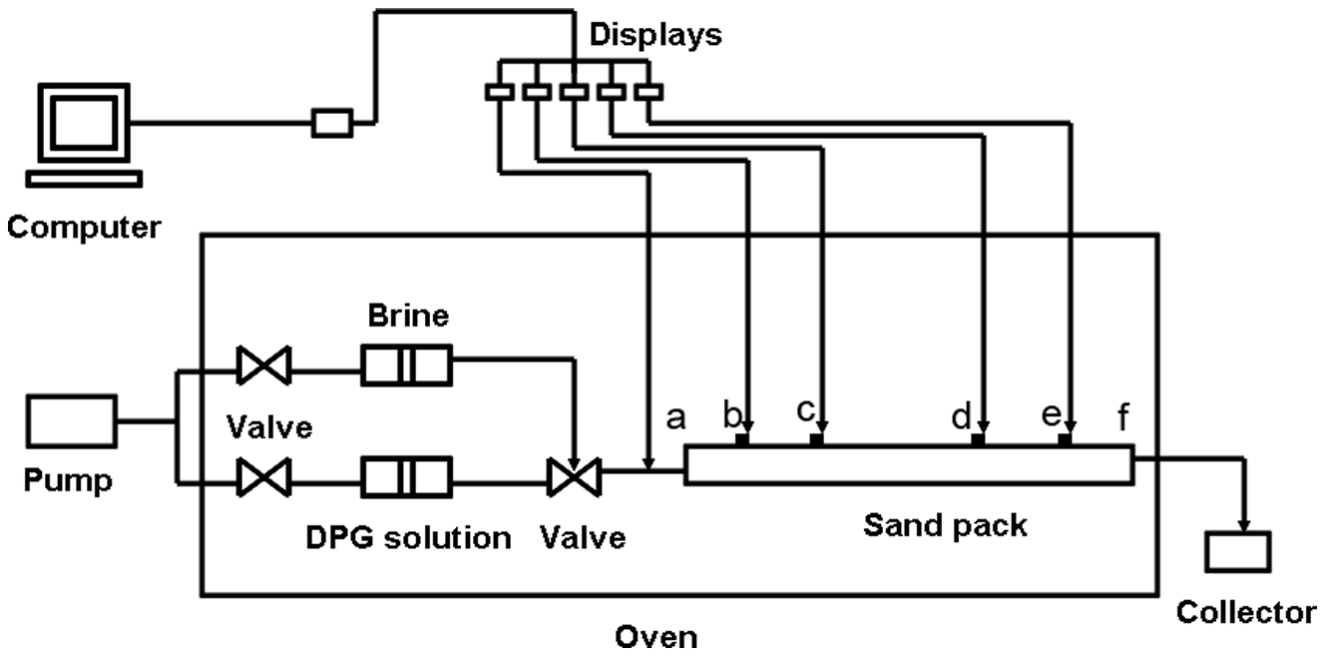
The multi-point pressure physical model.

The multi-point pressure physical model was studied to determine the injection capacity. The one-meter-long core has four internal pressure taps that divide the core into five sections (ab  =  bc  =  de  =  ef  = 10 cm, cd  = 40). The experimental flow chart is shown in Fig. 1. The steps for determining injection capacity are as follows: (1) Fill the pack with sand and weigh the dry core; (2) Saturate the core with brine and weigh the wet core, then calculate the pore volume; (3) Inject one pore volume (PV) of DPG particle solution into the core and place it into an oven at 75°C for 5 days; and (4) Flood water until the injection pressure reaches stability. In this research, the core permeability was 3.1 µm^2^ with a pore volume of 152 mL, and the injection concentration of DPG was 1600 mg/L.

### 2.4 Profile improvement capacity

**Figure 2:**
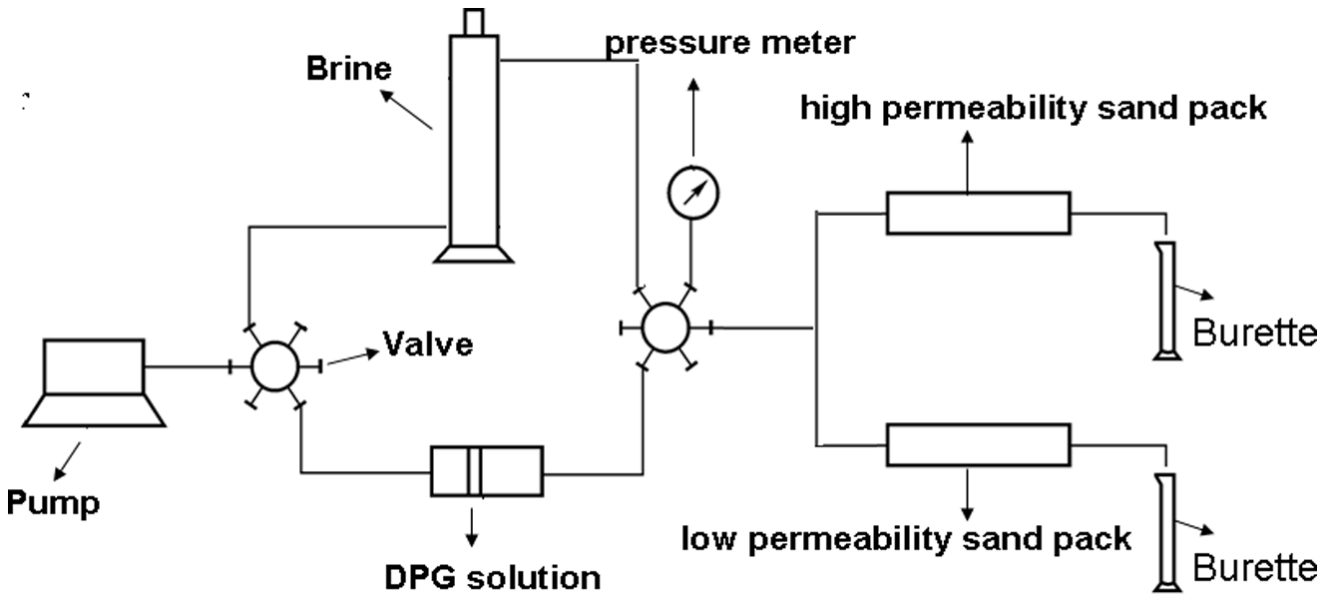
The double tube physical model.

The profile improvement capacity is determined by a double tube physical model (Fig. 2). In the experiment, the high permeability sand pack (permeability: 4 µm^2^) and the low permeability sand pack (permeability: 1 µm^2^) are used to simulate a heterogeneous formation. The pore volume was 30 mL, and the injection concentration of DPG was 1600 mg/L. When conducting this experiment, one pore volume of DPG solution was injected into the sand pack. Then, the sand pack was placed at 75°C for 5 days. Next, water flooding was performed until the pressure and produced fluid reached a stable condition. The fluid produced is recorded to calculate the profile improvement rate according to equation (1):
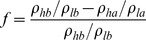
(1)where
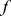
is the profile improvement rate, 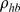
is the water-produced rate before the DPG solution injection in the high permeability sand pack,
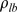
is the water-produced rate before the DPG solution injection in the low permeability sand pack, 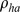
is the water-produced rate after the DPG solution injection in the high permeability sand pack, and 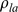
is the water-produced rate after the DPG solution injection in the low permeability sand pack.

### 2.5 Water and oil disproportionate permeability reduction capacity

**Figure 3:**
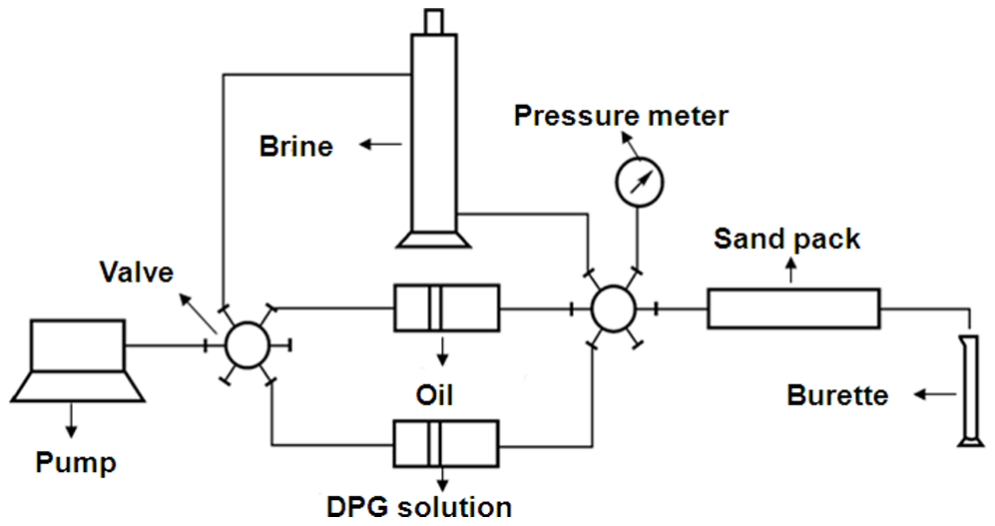
The single tube experimental model.

The single tube experimental model was carried out to evaluate the water and oil disproportionate permeability reduction capacity of the DPG particles. The experimental flow chart is shown in Fig. 3. The steps for determining the selective plugging capacity of water and oil are as follows: (1) Fill the pack with sand and weigh the dry core; (2) Saturate the sand pack with brine and weigh the wet core, then calculate the pore volume and water permeability (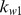); (3) Flood oil until no more water is produced in the sand pack model, and calculate the oil permeability (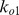); (4) Flood water until the pressure is stable and then calculate the water permeability (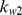); (5) Inject one pore volume of DPG solution into the sand pack, and place it into an oven at 75°C for 5 days; (6) Flood water again, and then calculate the water permeability (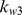); and (7) Flood oil again, and then calculate the oil permeability (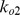). In this research, the residual resistance factor and elective index are used to illustrate the water and oil disproportionate permeability reduction capacity of the DPG particles [19]. The residual resistance factor equation (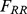) is calculated using the following equation (2):
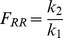
(2)where 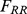 is the residual resistance factor, 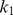
is the permeability before injection of DPG particles, and 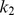
is the permeability after injection DPG particles.

The selective index (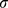) equation is
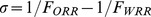
where 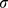 is the selective index ranging from 0 to 1. The closer the value is to 1, the stronger the disproportionate permeability reduction capacity is for water and oil. 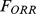 is the residual resistance factor of oil, and 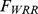 is the residual resistance factor of water.

### 2.6 Visual simulation experiment

**Figure 4:**
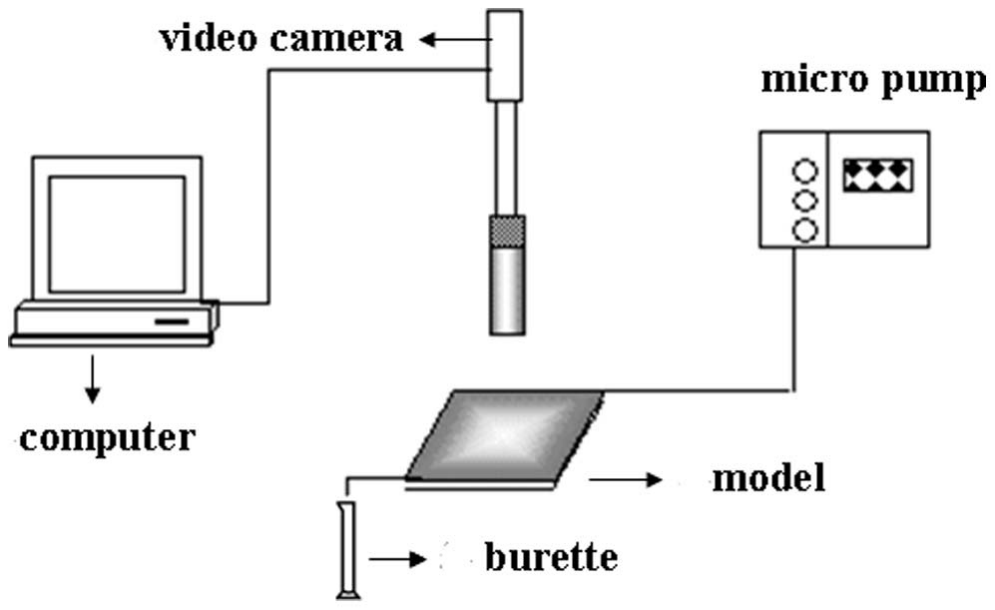
The visual simulation experimental flow chart.

Two visual simulation experiment models were used to investigate the profile control mechanism of the DPG particles: the flat panel sand model and the etched glass model. The flat panel sand model was filled with sand, and the size of this model was 25 cm×25 cm×1 cm. The etched glass model was prepared by laser etching, and channels with different sizes were distributed in the model. Both of the edges were sealed except two pores on the diagonal of the two models, which were used to simulate as an injection well and an oil well. The experimental flow chart is shown in Fig. 4. The steps were as follows: (1) Saturate brine in the model; (2) Saturate oil in the model; (3) Flood water until the effluent water cuts up to 98%; (4) Inject DPG solution into the visual model; and (5) Flood water until the effluent water cuts up to 98% again.

## Results

### 3.1 Preparation of DPG particles

**Figure 5:**
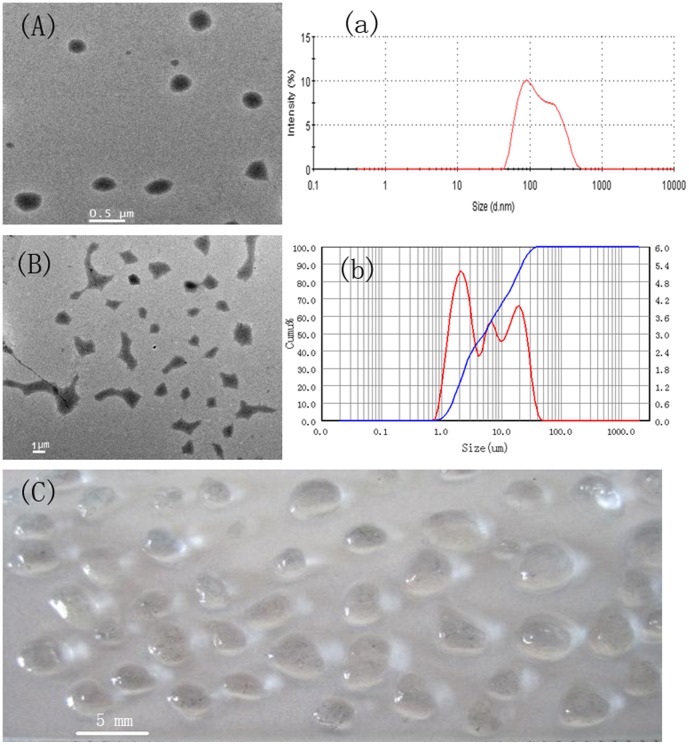
The morphology and size distribution of typical DPG particles. (A) The TEM morphology of nano-size DPG particles; (a) Nano-size DPG particles with an average size of approximately 108 nm; (B) The TEM morphology of micron-size DPG particles; (b) Nano-size DPG particles with an average size of approximately 5.6 µm; (C) mm-Size DPG particles with an average size of approximately 3.2 mm.

The DPG particles were prepared from a polymer gel at room temperature [17]. By adjusting the shearing time and rotational speed, the size distribution of DPG particles can range from nano- to micron- to mm-size. The morphology and size distribution of typical DPG particles are shown in Fig. 5.

Fig. 5 shows representative micrographs and the size distribution of the DPG particles. The rotational speed and shearing time in Fig. 5 (A), (B) and (C), are, respectively, set at 3000 rpm for 15 min, 2250 rpm for 6 min and 1000 rpm for 3 min. The morphology of the DPG particles is clearly presented in Fig. 5. The results demonstrate that these particles are relatively uniform, characteristic of a polygonal morphology. As the shearing time and rotational speed decrease, the size distribution of DPG particles increases, and the prepared DPG particles can be directly observed. The shape of DPG particles tends to diversify when increasing the size distribution. These diversified shapes can be attributed to the different parameters in the preparation process. In this research, the DPG particles were prepared using cross-linked gel systems through colloid mills at room temperature. When the bulk gel is pumped through a narrow gap that is formed by the rotating inner cone and the stationary outer cone, the shearing forces, generated from the relative movement between the stator and the rotor, are conducted in the bulk gel, which makes the bulk gel change into small particles. Shearing forces play a crucial role in the DPG particle preparation process. The longer the shearing time and the higher the rotational speed, the larger the shearing forces are. As a consequence, the larger shearing forces are conducted to the bulk gel, which forms DPG particles of nano-size or micron-size, in turn, forming DPG particles of mm size. In addition, the surfaces of the rotor-stator systems are not smooth but roughened and toothed, which increases wall friction and reduces slip and then forms polygonal particles.

However, for our DPG particle systems, the DPG particles have diversified shapes, which is more useful for injection into the formation. The formation contains grain and pore space where the fluid (water, petroleum) can be stored. In general, the pore space has fluid pathways that are tortuous, variably constricted, and usually highly connected [20]. Additionally, the pore shape and size are quite different. However, the pore spaces are connected and then form a high permeability channel after a long period of water flooding, resulting in invalid circulation of injection water and a low oil recovery. When injecting the DPG solution into the formation, the different shapes and sizes of DPG particles can be injected into different pores and channels, thus plugging pore throats in high permeability and reduce the permeability of the reservoir cores.

### 3.2 Injection capacity of DPG particles

**Figure 6:**
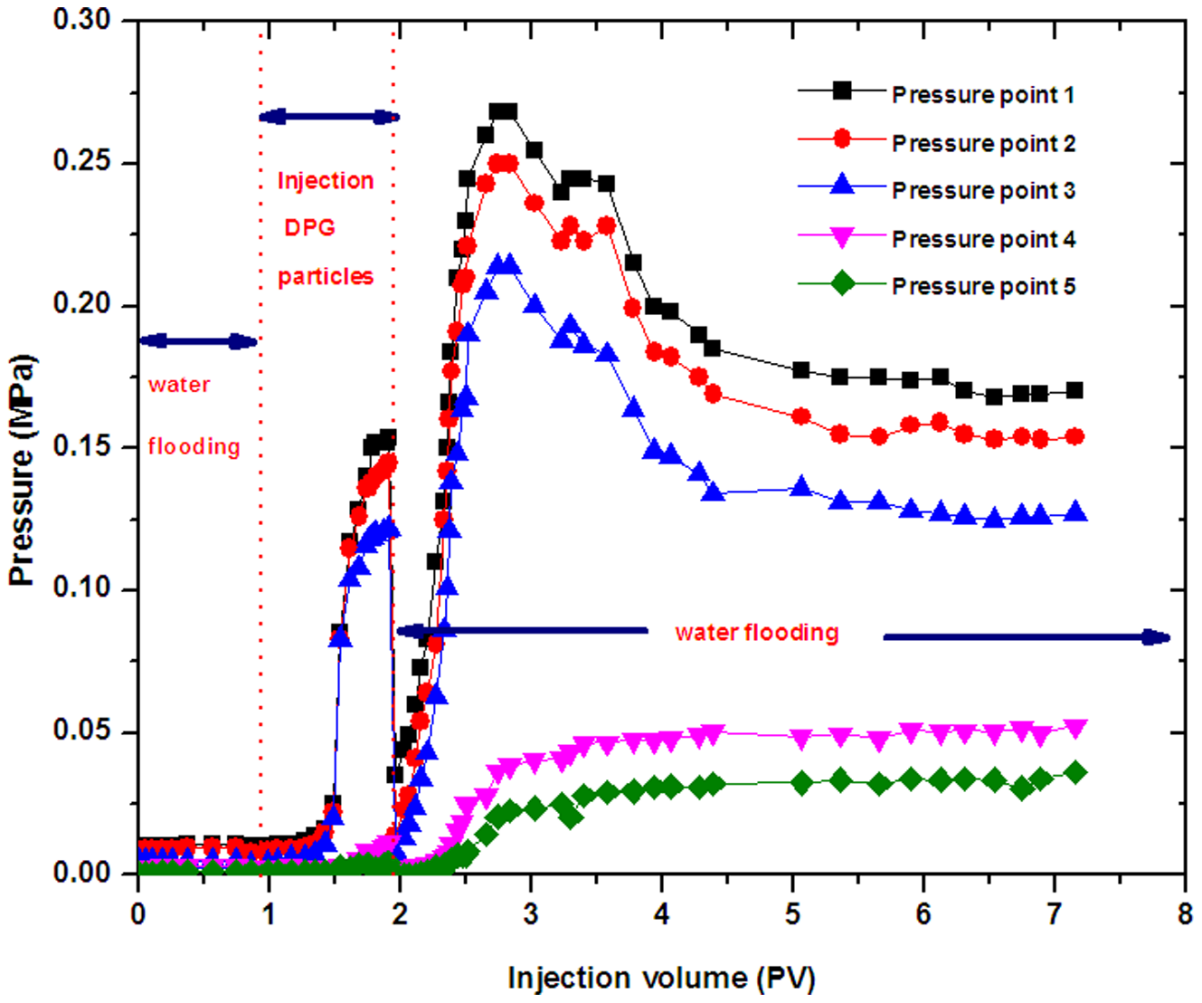
The pressure changes at different stages.

The micron size of DPG particles (average size: 5.6 µm) was used to investigate the injection capacity of the particles. Fig. 6 shows the injection capacity of DPG particles. The changes of pressure are a characteristic feature in the DPG particle injection process. The pressures decrease slightly at the initial water flooding stage, indicating that a high permeability zone was formed. When injecting the DPG particles into the sand pack, the pressures increase slowly at the initial stage, and the differences in the five pressure point values are not significant. However, the pressures increase more rapidly after injecting 0.42 pore volume of DPG particles. These five pressure points then reach the maximum values after injecting 1 PV of the DPG particle solution. The pressure changes can be explained as follows. First, a high permeability zone was formed after the long-term water flooding, resulting in invalid water circulation and pressure drop. When the DPG particles were injected, the high permeability zones were plugged and forced the injected fluid to turn to lower permeability zones, which makes the pressure increase. Additionally, due to the soft properties of DPG particles, they can be deformed and easily pass through pore throats, thus entering the in-depth formation. As a result, the pressures increase.

However, after 5 days of continuous aging at 75°C, pressures increase rapidly when water flooding begins again. The top pressures in the following water flooding stage are apparently larger than the top pressures in the injection stage. As the water flooding continues, the top pressures drop, but the pressures still maintain a high level after several pore volumes of water injection. The pressure changes can be explained as follows. The injected DPG particles have a relatively small negative charge, which is more prone to aggregate when injecting the particles into the formation at a high temperature. The metal ions (Na^+^, Ca^2+^, et al.) from the formation water will neutralize the negative charges of the DPG particle surface, so a decrease in the charge will result in a reduction in the electrostatic repulsions between the DPG particles. As a consequence, a large number of DPG particles bind into tiny microaggregates. Microaggregates, in turn, combine to form larger aggregates in the formation. The larger aggregates then bridge across the pore throats and reduce the permeability of the reservoir cores, resulting in a high pressure.

### 3.3 Profile improvement capacity

The profile improvement capacity serves as an important parameter in characterizing profile control treatments of DPG particles. In this initial stage of development, water flooding was usually used to exploit oil in the formation under homogeneous reservoir conditions. However, long term water flooding during the development of the oilfield has resulted in aggravated reservoir heterogeneity, with the injection water breakthrough into producing wells along the high permeability channels leading to invalid circulation of injection water and low oil recovery. In this case, it is necessary to improve reservoir heterogeneity through injection treatments of DPG particles.

**Figure 7:**
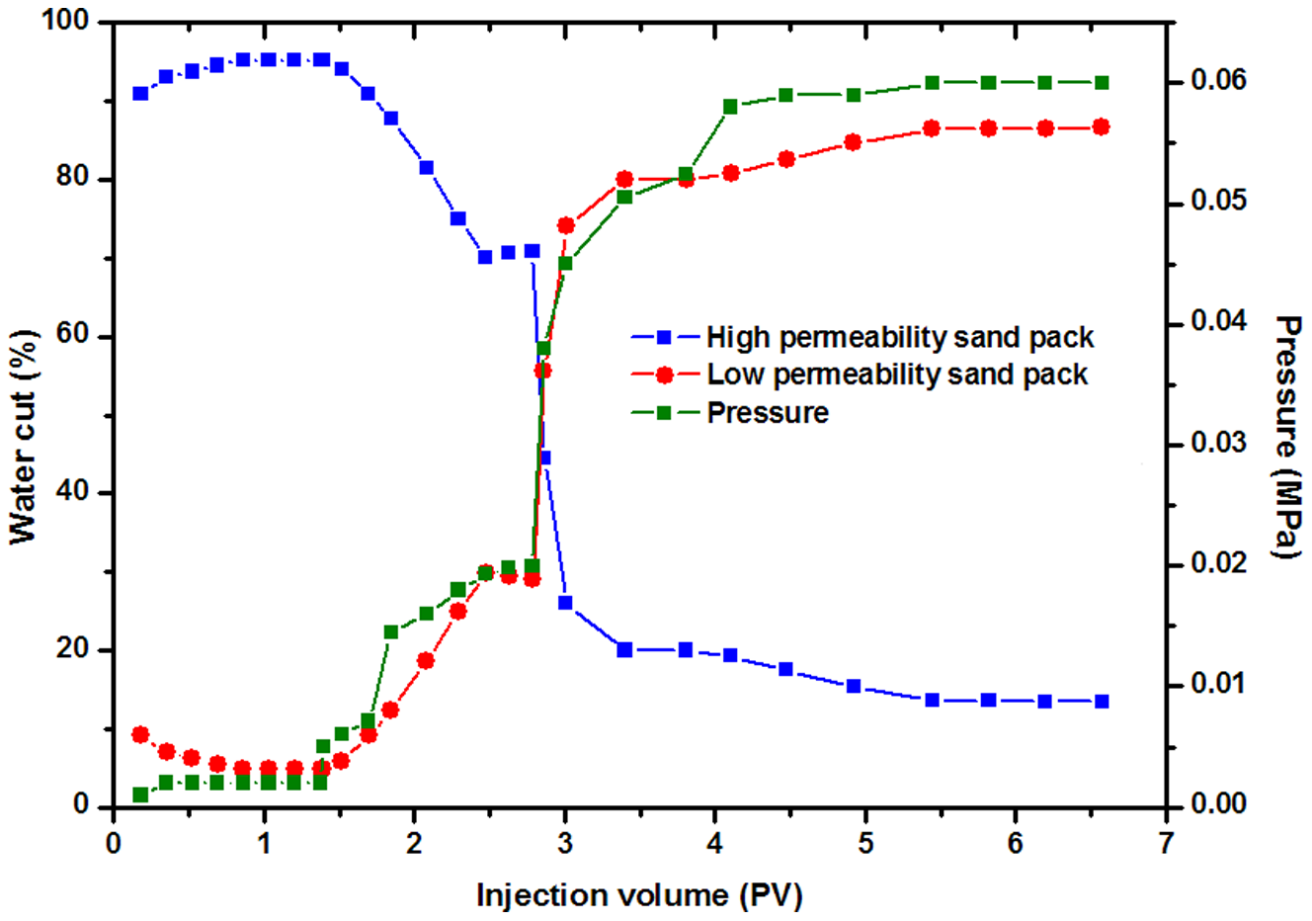
The profile improvement capacity of DPG particles.

Fig. 7 shows the pressure and water cut changes through the whole injection stage. During the water flooding stage, almost all of the injected water was produced from the high permeability physical model, and the water cut finally reached 98%, resulting in invalid circulation of injection water. In addition, the injection pressure was quite low at the water flooding stage. However, the injection pressure suddenly increased after injecting 1 PV of DPG particles. Meanwhile, more water was gradually produced from the low permeability physical model, especially after aging for 5 days at 75°C. According to equation (1), the profile improvement rate was 84.01%, demonstrating that injection particles can effectively improve the formation profile. When injecting DPG particles into physical models, the particles preferentially enter the high permeability physical model with retention in the core, which increases water flow resistance. Additionally, with high temperature aging, DPG particles combine into large aggregates that can bridge across the pore throats and effectively reduce the permeability of the porous medium in the high permeability zone. As a consequence, the injection water that follows can easily enter the low permeability physical model, and both the sweep efficiency and the formation profile are improved.

### 3.4 Disproportionate permeability reduction capacity

Table 1 shows the disproportionate permeability reduction capacity of the DPG particles. The results show that both water and oil permeability were reduced when DPG particles were injected, but the residual resistance factor of the water is significantly larger than the residual resistance factor of the oil, and the selective index reaches more than 0.3 after injecting DPG particles, indicating that the DPG particles have good disproportionate permeability reduction capacity for water and oil. As described above, the DPG particles were prepared using polymer gel systems through a high speed shearing method, with only physical shearing involved in the preparation process, so the disproportionate permeability reduction mechanisms of the gel can be used to explain the disproportionate permeability reduction effect of the DPG particles [19], [21], [22]. The segregated flow of oil and water pathways through a porous medium plays a dominant role in the disproportionate permeability reduction. During the high water fractional flow, water flows through most of the open pathways while some of the open pathways remain connected by oil and inaccessible to water. The entrance pressure for water may not be sufficient to penetrate the small pores, which remain open to oil after placement of the DPG particles. As a result, many oil pathways could remain connected and DPG particle-free after treatment. In this way, the DPG particles could reduce water permeability more than oil permeability. In addition, the DPG particles preferentially enter the high permeability channel, and stretching coiled macromolecules in the DPG particle layer under elongated flow could make the pore throats more constricted at higher water rates. Water flows will be restricted, but the oil flows cannot be restricted. As a consequence, the DPG particles reduce water permeability substantially more than they reduce oil permeability.

**Table 1:** Disproportionate permeability reduction of DPG particles.

No.	Items	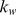 (water permeability,µm^2^)	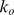 (oil permeability,µm^2^)	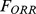	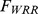	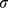 (selective index)
1	Before injecting DPG	0.849	0.119	---	---	0.356
	After injecting DPG	0.04467	0.0486	19.006	2.449	
2	Before injecting DPG	2.210	0.207	---	---	0.671
	After injecting DPG	0.367	0.173	6.022	1.195	
3	Before injecting DPG	4.850	1.191	---	---	0.451
	After injecting DPG	0.4620	0.6510	10.502	1.830	

### 3.5 Oil recovery in the visual model

The experiments, including flat panel sand model and etched glass model, were performed to illustrate the profile control of the DPG particles and the enhanced oil recovery mechanism.

#### 3.5.1 Oil recovery in the flat panel sand model

**Figure 8:**
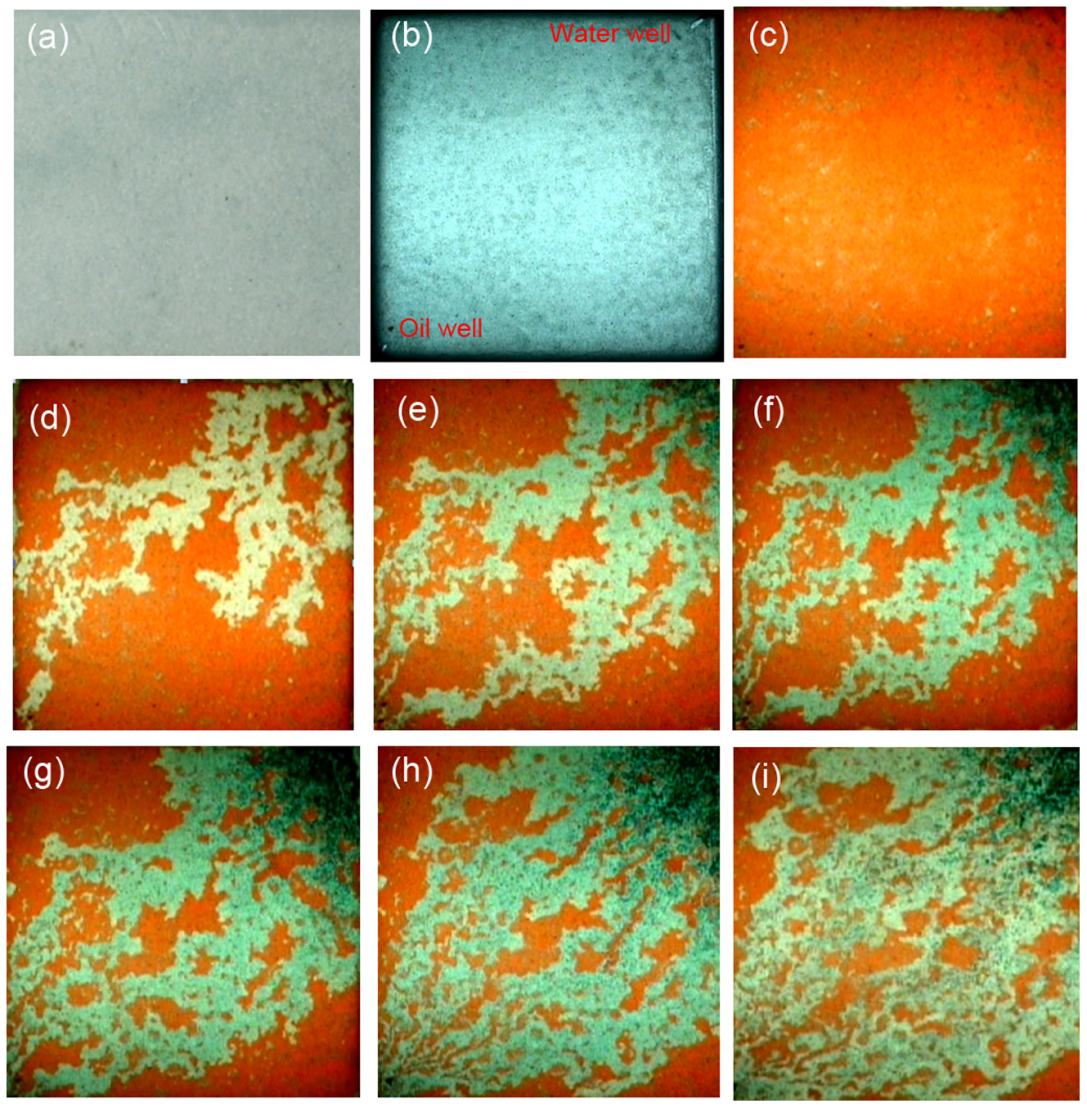
Visual simulation results in a flat panel sand model. (a) model; (b) saturating water; (c) saturating oil; (d) water flooding until water cut is up to 98%; (e) injection of DPG particles for 6 min; (f) injection of DPG particles for 9 min; (g) injection of DPG particles for 15 min; (h) injection of DPG particles for 20 min; (o) water flooding until water cut is up to 98% again.

Fig. 8 shows the whole process of using DPG particles as profile control treatments in the flat panel sand model. For ease of observation, the oil was dyed red, and the DPG particles were dyed green in this model. Fig. 8(c) simulated oil distribution in the original formation. In this initial stage of development, water flooding was usually used to exploit oil in the formation. However, a high permeability zone (Fig. 8(d)) was formed after a long period of water flooding, resulting in invalid circulation of injection water and low sweep efficiency. Oil recovery was only 27%, and a significant amount of oil still remained in the formation after water flooding. To enhance oil recovery, DPG particles were injected for the profile control treatment. The DPG particles preferentially entered the high permeability zone (Fig. 8(g)) and bridged across the pore throats, which can effectively increase water flow resistance, thus plugging high permeability zones. Fig. 8(e)∼(h) shows that the swept volume increases with increasing injection of DPG particles. When the water was re-injected, the water would encounter a greater flow resistance than before in the high permeability zone. As a result, the water was diverted into unswept low permeability zones, the sweep efficiency was improved and the residual oil was driven out of the small zones into the production well, leading to an enhanced oil recovery up to 49.3% (Fig. 8(i)).

#### 3.5.2 Oil recovery in etched glass model

**Figure 9:**
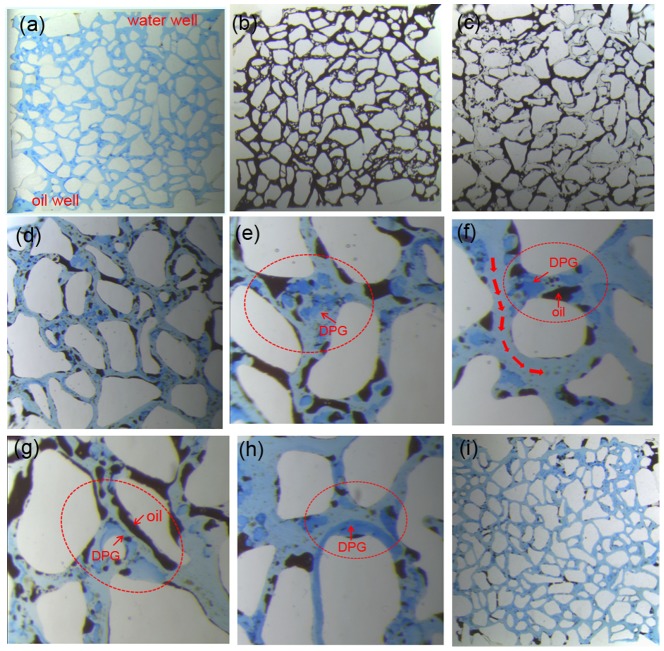
Visual simulation results in etched glass model. (a) model; (b) saturating water; (c: saturating oil; (d) water flooding until water cut is up to 98%; (e) retention in larger pore space; (f) directly plugging the small pore throat; (g) segregated flow of oil and water pathway; (h) adsorption on the surface; (o) water flooding until water cut is up to 98% again.

**Figure 10:**
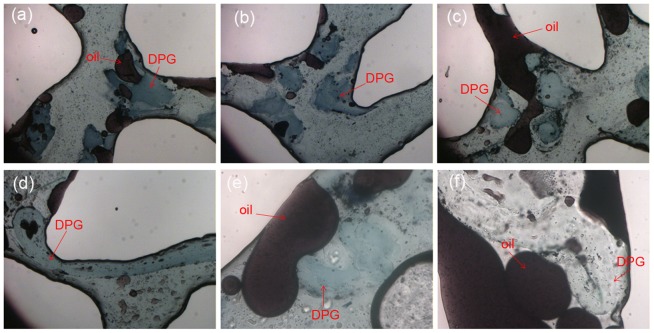
DPG particle distribution in etched glass model at different magnifications. (a)∼(d): 40× magnification; (e)∼(f): 100× magnification.

The etched glass model was used to simulate channel distribution under reservoir conditions, easily illustrating the profile control of the DPG particles and an enhanced oil recovery mechanism. In the model, the black fluid was oil, and the DPG particles were dyed blue. Fig. 9 shows the whole water flooding stage and DPG particle injection stage. Fig. 9 (c) shows that a large amount of oil still remained in the formation after the water flooding. So the residual oil remaining in the unswept zone is a prime target for enhanced recovery. When DPG particles are injected, the particles preferentially enter the high permeability channels (Fig. 9 (d)). Through retention in the larger pore space (Fig. 9 (e)), directly plugging the small pore (Fig. 9 (f)) or adsorption on the surface (Fig. 9 (h)), the DPG particles can block thief-zones in heterogeneous formations and divert trailing water-flooding fluid to adjacent low permeability zones, thus enhancing oil recovery. Additionally, Fig. 9 (h) shows a segregated flow of oil and water pathways that can confirm the oil flow in its own pathway. So the residual oil can easily be swept away when the injection pressure increases. To further understand the enhanced oil recovery mechanism of the DPG particles, experiments at 40× and 100× magnification were conducted to observe the distribution of the particles in the etched glass model (Fig. 10). When the pore size is smaller than the DPG particle size, the particles plug the small pore directly (Fig. 10(a)). However, when the pore size is larger than the DPG particle size, two or more particles will be stranded in the pore space and bridge onto the pore throat surface (Fig. 10(b) and (c)). Once the bridge is formed and consolidated, the newly arriving particles accumulate upstream from bridged pores, thus decreasing the following fluid flow rate and yielding fluid diversion effects. Additionally, the DPG particles can be deformed and pass through pores easily (Fig. 10(e)), so they can enter the in-depth formation and plug the high permeability channels. Moreover, the propagation of the DPG particles through pores will cause a negative pressure effect that contributes to carrying out the blind side residual oil or the residual oil attached on the rock surface, thus enhancing oil recovery. When the DPG particles are stranded in the pore space, a significant interface between oil and particles is formed (Fig. 10(d) and (f)). As the injection pressure increases, the residual oil can easily be stripped and driven out.

### 3.6 Enhanced oil recovery mechanism

**Figure 11:**
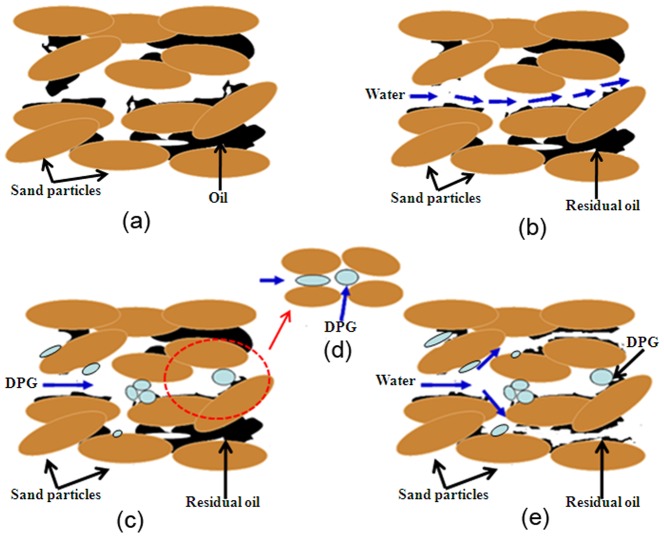
Schematic illustration for the enhanced oil recovery mechanism. (a) oil distribution in the initial stage of reservoir development; (b) a high permeability zone formed after long-term water flooding; (c) injection of DPG particles for profile control; (d) DPG particles deform and pass through pores; (c) water flooding after the treatment.

Based on the above research, an enhanced oil recovery mechanism for DPG particles has been proposed and is shown in Fig. 11. First, a high permeability zone was formed after long-term water flooding, resulting in an invalid circulation of the injection water, so a large amount of residual oil still remained in the formation (Fig. 11(b)). To enhance oil recovery, DPG particles were injected for the profile control treatment. When DPG particles were being injected, the particles preferentially entered the high permeability zone. DPG particles can pass directly or by deformation through porous media and enter the in-depth formation. By retention, adsorption, trapping and bridging, DPG particles can effectively reduce the permeability of the porous medium in the high permeability zone and divert fluid into the low permeability zone, thus improving the formation profile and enhancing oil recovery. When the size of the DPG particles is much larger than the pore throat size, the particles will be trapped at the entrance of pore throat and directly plug the high permeability channel. When the DPG particle size is a little larger than the pore throat size but not very much, the particles can be deformed and pass through the pore throat (Fig. 11(d)). Moreover, the deformable particles can revert to their original shape after entering a larger pore. Additionally, when the DPG particle size is smaller than the pore throat size, the particles pass directly through the pore throat and enter the in-depth formation. Subsequently, two or more particles will be stranded in the pore space and bridge onto the pore throat surface (Fig. 11(c)), which will effectively restrict pore space and decrease permeability, yielding fluid diversion into the low permeability zone (Fig. 11(e)). As a result, the formation profile is improved, and then the residual oil is driven out.

## Discussion

In the present report, the DPG particles with nano- to micron- to mm size were used for profile control treatment. Although the profile control and enhanced oil recovery mechanism, including injection polymers, gelling systems, particle systems, and foams, etc., have been studied, few studies have been conducted on DPG particles. The results demonstrate that DPG particles propagate through porous media by two patterns: direct pass and deformation pass. The “direct pass” pattern ensures that DPG particles have good injection capacity, whereas the “deformation pass” pattern makes DPG particles enter the in-depth formation. The propagation pattern is different from previous plugging agents such as polymers and gelling systems that can only directly pass through porous media without deformation. DPG particles and foams are easily broken when propagating through the pore throat. In addition, DPG particles preferentially enter the high permeability zone under heterogeneity reservoir conditions. By retention, adsorption, trapping and bridging, DPG particles can effectively reduce the permeability of a porous medium in a high permeability zone and divert fluid into a low permeability zone, thus increasing the swept volume of the fluid. Another important characteristic is the disproportionate permeability reduction effect in the DPG particle injection process, which results in the disproportionate permeability reduction of water and oil, and the effect is significant. The segregated flow of oil and water pathways through a porous medium plays a dominant role in disproportionate permeability reduction, similar to gel. By injection treatment with DPG particles, DPG particles can effectively reduce the permeability of the porous medium in a high permeability zone and divert fluid into a low permeability zone, thus improving the formation profile and enhancing oil recovery.
